# Natural Killer T Cells Subsets in Cancer, Functional Defects in Prostate Cancer and Implications for Immunotherapy

**DOI:** 10.3390/cancers3033661

**Published:** 2011-09-20

**Authors:** Michael Nowak, Ingo G.H. Schmidt-Wolf

**Affiliations:** Department of Internal Medicine III, University Hospital Bonn, Sigmund-Freud-Strasse 25, Bonn 53127, Germany; E-Mail: ingo.schmidt-wolf@ukb.uni-bonn.de

**Keywords:** NKT cell, prostate cancer, immunotherapy

## Abstract

Natural killer T cells are T lymphocytes with unique activation and effector properties. The majority of NKT cells, termed type-I or iNKT cells, recognize lipid antigens presented on MHC-like CD1d molecules. Type-I NKT cells have the capacity to rapidly secrete various cytokines upon activation, thereby regulate immune responses exerts dominant anti-tumor and anti-microbial effector functions. Specific activation of type-I NKT cells in mouse models boosts immunity and prevents metastasis, which has led to a number of phase I-II clinical trials. Since the discovery of NKT cells other subsets with different specificities and effector functions have been described. This article briefly reviews the physiological functions of NKT cell subsets, their implications in cancer and the attempts that have been made to employ NKT cells for immune therapy of cancer.

## 1. Natural Killer T Cells

Natural killer T (NKT) cells are a subset of innate lymphocytes with unique activation and effector properties. The majority of NKT cells (termed type-I NKT or iNKT cells) express a semi-invariant T cell receptor using the segments Vα14 in mice and Vα24 in humans rearranged with Jα18 segments and preferentially paired with Vβ38.2 and Vβ11 segments [1,2]. Recently, NKT cells expressing an invariant TCR comprised of the segments Vα10 and Jα50 have been identified [3].

Unlike conventional T cells, which recognize peptides embedded in MHC molecules, type-I NKT cells recognize lipid antigens presented in monomorphic, MHC-like CD1d molecules [4,5]. Type-I NKT cells are CD1d-restricted, hence mice lacking CD 1d molecules or associated beta2-microglobulin lack these cells [6]. Upon TCR-mediated activation type-I NKT cells produce various cytokines, of which some may have opposite functions. Secreted cytokines include both regulatory factors (e.g., IL-4, IL-13, IL-10, TGF-β) as well as those with a clear pro-inflammatory function (e.g., IL-2, IL-17, IFNγ, TNF-α) [7-9]. Naming feature of NKT cells is their expression of typical markers of natural killer (NK) cells. These proteins include both inhibitory and activating killer receptors (including NK1.1 through which NKT cells can exert cytotoxic effector functions [10]. Nonetheless, most attention has been attributed to the capacity to rapidly release different cytokines. Hence, type-I NKT cells were shown to contribute to a variety of different biological systems such as host defense against pathogens, tumor immune surveillance and immune tolerance [11-14].

The prototypic ligand for type-I NKT cells, α-galactosylceramide (α-GC), has been identified from a screen for marine compounds with anti-cancer effects [15]. In a number of studies, which will be discussed later, α-GC administration in mice prevented tumor metastasis [16]. Subsequently, several type-I NKT cell-activating CD1d ligands derived from pathogenic and non-pathogenic micro-organisms have been identified [17-21]. However, the identity of an endogenous ligand for type-I NKT cells remains elusive [22-25]. Finally, it has to be noted that in the absence of CD 1d stimulation type-I NKT cells can be activated by combinations of cytokines, such as IL-12 and IL-18 [26,27]. Type-I NKT cells activated by CD1d: α-GC complexes secrete IL-4 within minutes after activation, which is followed by a sustained secretion of IFNγ, displaying opposite biological functions to IL-4. This bi-functional cytokine secretion profile has led to the development of several α-GC modifications stimulating a pronounced Th1 cytokine profile and thus increased anti-tumor activity observed in murine cancer models [28-31].

The main CD1d-expressing cell types were identified as dendritic cells (DC), macrophages, and B cells. Physiological functions of CD1d molecules have intensively been analyzed in the case of DC. Interactions between NKT cells and DC differ in some key features of those between classical T cells and DCs. Type-I NKT cells constitutively exhibit a memory phenotype and thus do not require priming. Activation of type-I NKT cells and IFNγ secretion follows contacts between CD 154 (CD40L)–CD40 and CD80/86 to CD28 molecules and elicits IL-12 secretion in DCs which stimulates IFNγ secretion in NKT cells. These interactions explain the marked ability of type-I NKT cells to mature DCs and amplify immune responses and is consistent with the requirement for type-I NKT cells for low-dose IL-12 immunotherapy in some anti-tumor responses [32-34]. Some cancer types also express CD1d, including prostate, glioma, hepatocellular carcinoma, B-CLL, and multiple myeloma, suggesting NKT cells can directly interact with tumors [35-38]. Recent studies indicate that tumor cells expressing CD1d may present lipid antigens thereby bias the effector functions of type-I NKT cells towards tolerance. For instance, prostate tumor cells by expressing CD1d molecules inhibit the activation of IFNy secretion by type-I NKT cells [38]. Sriram *et al.* showed that pharmacological blockade of glycolipid shedding from the cell surface of a lymphoma cell line rescues the recognition and killing of such cells by type-I NKT cells [39]. Along this line, NKT-mediated killing of early stage myeloma cells which express CD1d molecules is lost upon transition to advanced myeloma stage and subsequent loss of CD1d expression [40].

## 2. Type-I NKT Cell Activities in Cancer

Type-I NKT cells were shown to contribute to immune surveillance in spontaneous and carcinogen-induced cancers. Mice deficient in Jα18 or CD1d lack type-I NKT cells and were found to be more susceptible to methylcholanthrene-induced carcinoma [41]. Numerous studies demonstrated cancer-related type-I NKT cell defects in various types of human cancer, including advanced prostate cancer, multiple myeloma, melanoma, colon, lung, and breast cancer [42]. Despite overall variations of peripheral blood type-I NKT cells between 10–1,000 NKT cells/million T cells in healthy individuals, numbers of type-I NKT cells in cancer patients were consistently decreased [43]. Those NKT cells remaining in the circulation were refractory to α-GC-stimulated IFNγ secretion accompanied with a diminished proliferation capacity. Reminiscent of conventional T cells, IL-2 was sufficient to reverse the block in proliferation of NKT cells *in vitro*. Diminished IFNγ responses observed in multiple myeloma and prostate cancer patients could be reversed by co-administration with α-GC and IL-12 administration, respectively [37,44]. Comparable to the situation in humans, decreased NKT numbers and defective functions were observed in several murine tumor models [38,45].

## 3. Type-I NKT Cells in Prostate Cancer

Tahir *et al.* first described numerical and functional type-I NKT cell defects in advanced prostate cancer patients [44]. Similar defects were later found in the murine transgenic adenocarcinoma of the mouse prostate (TRAMP) model [38]. Consistent with this, Bellone *et al.* demonstrated the exacerbation of prostate cancer in type-I NKT cell-deficient TRAMP mice [45]. TRAMP mice are transgenic for the SV40 large T antigen (Tag) under control of the rat Probasin promotor. Beginning with puberty, male TRAMP mice express the oncogene and progressively develop prostate intraepithelial neoplasia as early as age of 10 weeks. TRAMP tumors metastasis spreading to lymph nodes, lung, and bone marrow, thus exhibit histological features of human prostate cancer [46].

**Figure 1.**
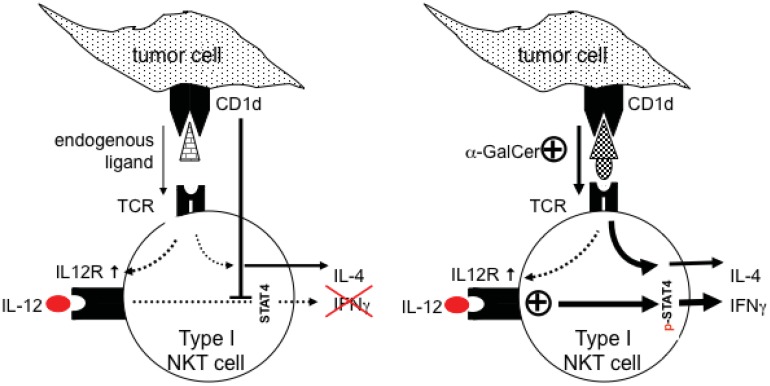
Proposed model of NKT cell-tumor interactions in murine prostate cancer.

We characterized the interactions between type-I NKT cells and tumor cells in this mouse model ([38], Figure 1). Upon α-GC administration serum levels of the cytokines IL-4, IFNγ as products of iNKT cells as well as IL-12 as a product of activated DCs were diminished in tumor-bearing mice, suggesting type-I NKT cells were refractory to stimulation. The tumor cell line TRAMP-C2 [47], human prostate tumor cell lines as well as mouse prostate epithelium (PrEC) expressed CD1d molecules on the surface, suggesting prostate (tumor) cells can directly interact with iNKT cells. Type-I NKT cells of healthy mice express low levels of the activation markers CD25, CD69, IL-12R in the steady state. Upon contact to TRAMP-C2 cells iNKT cells up-regulated these molecules and secreted IL-4. Notably, neither loading of tumor cells with α-GC nor addition of IL-12 were sufficient to induce the IFNγ production of NKT cells in contact to prostate tumor cells. Collectively, these data suggested that tumor cells, although up-regulating activation markers on type-I NKT cells (in particular the IL-12 receptor) inhibit complete responses, observed as a lack of IFNγ production. Only the combination of the high-affinity ligand α-GC plus IL-12 led to the secretion of IFNγ in healthy type-I NKT cells. Moreover, TRAMP-C2 cells inhibited the phosphorylation of the transcription factor STAT4, showing that tumor cells concurrently provide positive signals for activation (IL-12R up-regulation) and inhibit intracellular signals downstream of the IL-12R *(i.e.*, STAT4).

Which factors are responsible for the IL-12R blockade is not fully clear. One may speculate that CD1d expressing prostate tumor cells present an Th2-biasing endogenous lipid antigen in CD1d molecules, explaining the basal production of cytokines in the absence of exogenous α-GC. Reminiscent of these data, Chang *et al.* isolated the glycolipid lysophosphatidylcholine (LPC) from plasma of multiple myeloma patients binding to CD1d and skewing the cytokine secretion of type-I NKT cells towards IL-13 [48].

Promising data of α-GC and NKT cells obtained from animal models led to a number of phase I and phase II clinical trials in cancer patients. These published and ongoing trials employed different approaches, sometimes used in combination (Table 1):

- Activation of endogenous type-I NKT cells by α-GC;
- Activation of endogenous type-I NKT cells by DCs/ monocytes, loaded with α-GC;
- Expansion and re-infusion of type-I NKT cells.

Giaccone *et al.* in a phase I study published in 2002 described the first experience with intravenously injected free α-GC into 24 patients with advanced cancer [49,50]. Frequencies of type-I NKT cells in patients were significantly lower compared to healthy individuals and further decreased to undetectable levels 24 hrs post-injection. Upon activation murine type-I NKT cells down-regulate TCR and NK markers for several days continuing to produce cytokines [51], hence, a decrease in detectable type-I NKT cells as Giaccone *et al.* observed might be judged as successful NKT cell activation. This notion has been challenged by other studies observing increased numbers of type-I NKT cells upon treatment [52,53]. In contrast to α-GC injection into mice, no liver toxicity could be observed in this study [49,50]. This might be attributed to the low number of type-I NKT cells resident in human livers compared to mice whose livers are naturally enriched for type-I NKT cells ([54], Table 2). Immunological effects as transient increases in GM-CSF and TNF-α serum levels were dependent on pre-treatment NKT numbers rather than α-GC dosage. Despite the decrease in NKT cell numbers and increases in serum cytokines no anti-tumor responses were observed in this study.

Preclinical data obtained from mice indicated that injection of α-GC loaded DCs results in prolonged secretion of cytokines and less pronounced TCR downregulation in comparison to injection of free glycolipid [55]. These observations led to several phase I-II studies in advanced cancer patients, improving the immunological and clinical outcome as compared to the initial study by Giaccone *et al.* (Table 1). Nieda *et al.* enrolled a total of 12 patients who received intravenously injected autologous, immature DCs loaded with α-GC [56]. Serum IFNγ levels significantly increased after the first DC administration and were further elevated after the second round of treatment, interpreted as a memory effect on NKT cells. Furthermore, two of 12 patients treated showed decreased serum tumor markers for up to 12 months post-treatment. Although transient drops of type-I NKT frequencies were observed after DC administration the overall frequencies did not change during the study course, suggesting that α-GC-loaded DC overcome functional defects of endogenous pre-treatment type-I NKT cells. Injected DCs mostly migrated to the liver and to lesser extent to the spleen of patients. In view that human liver harbors relatively low numbers of type-I NKT cells, specifically targeting DCs to spleens by using functionally mature DCs might improve the treatment outcome.

Mature DCs have been shown to be more potent in stimulating T cells and thus were expected to be superior NKT stimulators *in vivo*. Chang *et al.* intravenously treated five advanced cancer patients with α-GC-loaded monocyte-derived DCs subsequently matured by TNF-α, IL-1β and IL-6 [53]. Importantly and in contrast to other studies using free α-GC or α-GC loaded immature DC, administration of mature DC significantly increased and could be detected for up to six months post-treatment. Whether the increased basal IL-12 release by mature DCs or the altered migration (e.g., to spleens) accounts for the improved immunological effects remains unsolved.

Further published and ongoing trials were initiated to test the safety and therapeutic potential of *ex vivo* expanded and re-infused NKT cells. Kunii *et al.* designed a phase I trial in patients with recurrent head and neck squamous carcinoma received enriched iNKT cells and α-GC loaded DCs. Type-I NKT cells were injected into tumor-feeding arteries and DCs co-administered by nasal submucosal injection. Vα24^+^ NKT (type-I) cell numbers increased in seven of eight patients, three cases showed a partial response, four exhibited a stable disease [57].

## 4. Subsets of NKT Cells in Anticancer Therapy

NKT cells exhibit a significant heterogeneity in terms of specificity. Whereas type-I NKT cells constitute the majority of the NKT family and protect from tumor growth, at least three other subsets of NKT cells with different specificities, phenotypes and functions emerged, of which two were described in the context of immune surveillance [58] and will be discussed further.

### 4.1. Type-II NKT Cells

Type-II NKT cells are CD1d-restricted but express a polymorphic TCR. This cell type was first described by Cardell *et al.* demonstrating type-II NKT respond to different lipids than type-I NKT cells [59]. The main ligand of type-II NKT cells identified so far is sulfatide, a glycolipid derived from myelin sheath [60]. CD1d tetramers loaded with sulfatide or α-GC showed non-overlapping staining, suggesting that at least two distinct populations of natural killer T cells exist [59].

Type-II NKT cells were shown to suppress immune surveillance against different murine tumor models, including fibrosarcoma, colon carcinoma, renal carcinoma and B cell lymphoma [61-64]. Using an elegant system Ambrosino *et al.* reciprocally activated type-I and type-II NKT cells by α-GC and sulfatide, respectively, in tumor-bearing mice. Whereas activation of type-I NKT cells protected against tumor growth, as expected, activation of type-II NKT cells suppressed this protective effect [63]. Blockage of immune surveillance was dependent on the expression of IL-13, which subsequently induced Gr-1^+^ CD11b^+^ myeloid suppressor cells producing TGF-β [65]. Exceptionally, in the osteosarcoma model, Terabe *et al.* also provided evidence that type-II NKT cells can suppress immune surveillance independent of IL-13 [64]. Even more so, when type-I and type-II NKT cells were simultaneously stimulated with their respective CD1d ligands α-GC and sulfatide type-II NKT cells were able to suppress type-I NKT cells in a cell-cell contact dependent manner [63]. Whether (CD1d^+^) tumor cells present type-II NKT–stimulating ligands remains an unsolved, challenging question. A further unsolved issue is whether type-II NKT cells migrate into tumors and perhaps inversely correlate with disease state. To our knowledge, no clinical trials targeting type-II NKT cells have been conducted to date.

### 4.2. Cytokine-Induced Killer (CIK) Cells

A subset of lymphocytes showing a NKT cell-like behavior, termed cytokine-induced killer (CIK) cells, provided encouraging results in clinical studies in both autologous and allogeneic context [66,67]. CIK cells are a heterogenous population of cytotoxic T lymphocytes which express a non-invariant TCR repertoire, in the majority express the CD3^+^ CD56^+^ phenotype and show marked expression of the activating natural killer cell receptor NKG2D (CD314) and CD94. Unlike for type-I and type-II NKT cells the nature of CIK cell antigens remains elusive. CIK cells are generated *ex vivo* by incubation of peripheral blood lymphocytes with an agonistic anti-CD3 monoclonal antibody, IL-2, IL-1β and IFNγ [68]. CIK cells can be generated from CD1^−/−^ mice suggesting that these cells differ from type-I and type-II NKT cells [69]. Target recognition and cytotoxicity of CIK cells is non-major histocompatibility complex-restricted but NKG2D-dependent [70]. The expression of NK markers on CIK cells is reminiscent of virus-specific CD8^+^ T lymphocytes which acquire expression of inhibitory NK cell markers and thereby regulate the immune response in infection [71,72]. Even more so, recent data suggest that CIK cells are effector memory T cells [73].

Although CIK cells have been reported to express NK killer receptors, the exact mechanisms of tumor cell recognition remain under debate. Killing of AML cells by CD3^+^ (CD56^+^ and CD56^−^) CIK cells is dependent on interactions between HLA expressed on tumor cells and TCR but independent of NK cell receptors [74]. In contrast, Marten *et al.* demonstrated the improved anti-tumor activity of CIK cells to be mainly attributed to the pronounced proliferation rate leading to an increase in total lytic units [75].

Recently, DCs transduced with the tumor-associated antigen (TAA) PSMA were shown to stimulate CIK-cell mediated lysis of PSMA-expressing prostate tumor cells. Furthermore, the co-cultivation of Ad-PSMA-transduced DCs with CIK cells increased the production of IFNγ after restimulation with PSMA peptide mixtures [76]. In a recent study by Pang *et al.* the antitumoral effects of allotumour RNA-transfected DCs cocultured with autologous CIK cells on hormone-refractory prostate cancer were evaluated [77]. The cocultured cells significantly inhibited tumor growth in SCID mice and induced cancer cell necrosis and apoptosis. Maturation of tumor RNA-pulsed DCs with autologous CIK cells enhanced antitumor immunity, which could be induced by increased CD4^+^ Th1 and CD8^+^ T cells and decreased CD4^+^ CD25^+^ regulatory T cells.

Recently, clinical trials aimed at combining active immunotherapy using tumor vaccines with passive immunotherapy using CIK cells have been performed. Evidence is rising that the application of CIK cells in combination with pulsed DC may indeed improve the immune response towards cancer. Autologous CIK cells modified to produce IL-2 have been tested in patients with metastatic renal cell carcinoma, colorectal carcinoma and lymphoma in a phase I trial without major side effects [78]. In this study, ten patients received 1–5 intravenous infusions of IL-2-transfected CIK cells. While six patients remained in progressive disease, three patients showed no change by additional treatment, and one patient with lymphoma developed a complete response. Various trials using CIK cells have been successfully performed. Recently, a first report of the international registry on CIK cells summarized published trials [66]. In 11 trials CIK cells were adoptively transferred to 426 patients with various cancer entities including hepatocellular carcinoma, gastric cancer, Hodgkin and non-Hodgkin's lymphoma. In 384 patients a clinical response was reported, 24 of them showed a complete response, 27 patients a partial and 40 patients a minor response. The total response rate was 91/384 (23.7%) patients, 161 (41.2%) patients had a stable disease. Taken together, adoptive immunotherapy with CIK cells can prevent tumor recurrence and improve quality of life and progression-free survival. Meanwhile 596 patients with CIK cell transfusions have been reported [66].

## 5. Future Perspectives

Natural killer T cells have been acknowledged as potent regulators of immune surveillance. Despite promising preclinical data obtained in murine tumor models, first clinical trials on cancer patients administering free α-GC showed only modest effects. Significant improvements in terms of clinical responses have been made harnessing *ex vivo* expanded NKT cells in conjunction with autologous dendritic cells. A general drawback of these trials still is the discordance between the sites of biological action *(i.e.*, the tumor site) and the frequency and cytokine production of circulating type-I NKT cells as common read-out parameters. Whereas type-I NKT cells in most cases were administered intravenously, tumors are the site of biological action. A potent treatment approach has been designed by Kunii *et al.* by targeting type-I NKT cells to the tumor site by injection into tumor-feeding arteries [52]. To our knowledge, no information is available so far on the localization of NKT cells to and cytokine secretion at tumor site.

Further improvement of clinical trials which resulted only transient clinical responses so far has been hampered by the a lack of data about whether *in vivo* expanded NKT cells underly similar functional defects as endogenous NKT cells.

Since its discovery, the NKT cell family gave rise to several new subsets with different biological functions. Ambrosino *et al.* by demonstrating that type-II NKT cells inhibit the activation of type-I NKT cells opened up for a new field of investigation [63]. Future pre-clinical studies are required to elucidate the role of type-II NKT cells, which antigens they recognize in and how these subsets interact in human cancer.

## Figures and Tables

**Table 1.** Examples of published and ongoing clinical trials using NKT cell subsets.

**Indication**	**Treatment**	**Responses**	**Ref.**
**type-I NKT cells**			
advanced cancer	α-GC i.v.	no clinical response in 24/24, SD 1 in 7/24 patients	[50]
non-small cell lung cancer	α-GC-loaded PBMC, i.v.	increased serum IFNγ in 10/17, SD in 5/17, progressive disease in 12/17 patients	[79]
metastatic malignancies	α-GC-loaded monocyte -derived immature DC	increased IL-12 and IFNγ levels in 6/9 patients. Clinical responses: not monitored	[56]
multiple myeloma	α-GC-loaded monocyte -derived mature DC	Increased NKT numbers and serum IL-12 & IFNγ levels in 5/5 patients.	[53]
head and neck cancer	α-GC-loaded monocytes, intranasal submucosa	increased NKT numbers in 4/9, increased NK cytotoxicity in 8/9 patients	[52]
non-small cell lung cancer	α-GC-loaded monocytes, intranasal submucosa; type-I NKT cells i.a. (tumor-feeding)	increased NKT numbers in 7/8, PR 2 in 3/8, SD disease in 4/8 patients	[57]
metastatic malignancies	*in vitro* expanded type-I NKT cells, i.v.	Ongoing trial (NCT00909558)	
non-small cell lung cancer	*in vitro* expanded, activated type-I NKT cells, i.v. Dose-escalating.	No clinical response in 6/6 patients. Increased NKT numbers and increased IFNγ levels in 2/3 patients with level 2 dose of iNKT cells	[80]
melanoma	*in vitro* expanded type-I NKT cells i.v.	Ongoing trial (NCT00631072)	
**CIK cells**			
metastatic malignancies	IL-2 transfected CIK cells	progressive disease 6/10, CR 3 in 1/10 patients, SD ^1^ in 3/10 patients	[78]
non-small cell lung cancer	chemotherapy plus CIK cells	59 patients. Median survival time increased from 11 to 15 months	[81]
non-small cell lung cancer	activated CIK cells	42 patients. Increased 2-year survival rate (94.7±3.6% *vs* .78.8±7%)	[82]
renal cancer	CIK cells	CR in 3/16, PR in 1/16, SD in 6/16 patients	[83]

1 SD, stable disease;

2 PR, partial response;

3 CR, complete response.

**Table 2.** Functional differences between human and mouse NKT cells.

	**Human**	**Mouse**
Coreceptor expression	CD4^+^, CD8^+^, DN type-I NKT subsets	CD4^+^, DN type-I NKT cell subsets
type-I NKT cell cytokine profile	Th2 cytokines: CD4^+^ > CD4^-^	Less pronounced dichotomy of type-I
	Th1 cytokines: CD4^+^ < CD4^-^	NKT cell cytokine production
type-I NKT frequency (blood)	Blood: <0.1–1%	Blood: ∼1–2%
NKT cell distribution	Liver enriched for type-II NKT	Liver enriched for type-I NKT cells
Effects of α-GC injection	No anti-tumor response	Prevents tumor regression and metastasis
	No liver toxicity	Liver toxicity

## Abbreviations

α-GC: alpha-galactosylceramide
APC: antigen presenting cells
IL: interleukin
IFNγ: interferon-gamma
NKT: natural killer T cell
TCR: T cell receptor
DC: dendritic cells
CIK: cytokine-induced killer cells
